# Transient brachial plexus injury after robot-assisted elastic intramedullary nailing of midshaft clavicle fracture: a case report and literature review

**DOI:** 10.3389/fsurg.2025.1725023

**Published:** 2025-11-21

**Authors:** Zhongyu Wang, Zhiyao Zhao, Yufu Zhang, Xigong Zhang, Xiao Han, Yanchao Li, Maoqi Gong, Qiang Huang, Jie Tan

**Affiliations:** 1 Department of Orthopedic Trauma, Beijing Jishuitan Hospital, Capital Medical University, Beijing, China; 2 Laboratory for Clinical Medicine, Capital Medical University, Beijing, China; 3 Department of Orthopedic Surgery, Beijing Jishuitan Hospital, Peking University Fourth School of Clinical Medicine, Beijing, China; 4 Department of Rehabilitation Medicine, Beijing Jishuitan Hospital, Capital Medical University, Beijing, China

**Keywords:** clavicle fracture, brachial plexus injury, conservative treatment, case report, robot-assisted surgery

## Abstract

**Background:**

Although brachial plexus injury following clavicle fractures is rare, it can be a serious complication with significant implications for recovery. In this report, we present a case of midshaft clavicle fracture treated with elastic intramedullary nailing, where the patient developed brachial plexus injury symptoms two months post-surgery.

**Case presentation:**

A 37-year-old woman with a middle-third clavicle fracture presented to the emergency room 5 days after a traffic accident. The patient underwent robot-assisted minimally invasive elastic intramedullary nail fixation for the left clavicle fracture with TiRobot assistance. 2 months after surgery, the patient reported numbness in all fingers of the left hand, affecting both the palmar and dorsal sides, with a pronounced impact on the thumb. Additionally, the patient was unable to move the shoulder and elbow actively (grade 1). x-ray and CT imaging revealed significant callus formation at the fracture site. Electromyography (EMG) and Doppler ultrasound all suggested left incomplete brachial plexus injury with root-level impairment. Conservative treatment, including regular physiotherapist-supervised rehabilitation, was initiated. Over time, the patient experienced gradual improvement in muscle strength and resolution of numbness. 6 months after the onset of brachial plexus injury, the elastic intramedullary nail was removed. x-ray imaging revealed notable bone remodeling with reduced callus formation at the fracture site compared to the findings observed at the onset of the brachial plexus injury. The patient reported an overall recovery of 95% compared to her condition at the time of the injury, reflecting a highly satisfactory outcome.

**Conclusions:**

This case highlights the importance of vigilance for brachial plexus injury in patients with clavicle fractures and demonstrates the potential for successful outcomes with conservative treatment. Furthermore, it contributes to the limited body of literature regarding the rare occurrence of brachial plexus injury after elastic intramedullary nailing of clavicular fractures.

## Introduction

Clavicle fractures are common, accounting for 2.6%–4% of all fractures and 35%–44% of shoulder girdle fractures (1, 2). Generally, fractures with minimal displacement can be effectively managed conservatively, often resulting in satisfactory outcomes. However, for fractures with significant displacement or open injuries, surgical intervention is preferable. Previous studies have reported complications such as fracture malunion, vascular injury, and pneumothorax; however, brachial plexus injury is extremely rare (3–6). Additionally, the onset and presentation of compressive symptoms vary, with some patients experiencing sudden onset while others have delayed symptom onset, complicating diagnosis and often leading to missed cases (7).

In this report, we present a case of midshaft clavicle fracture treated with elastic intramedullary nailing, where the patient developed brachial plexus injury symptoms two months post-surgery. This report aims to highlight the rare complication of brachial plexus injury following clavicle fractures.

## Case presentation

**Figure 1:**
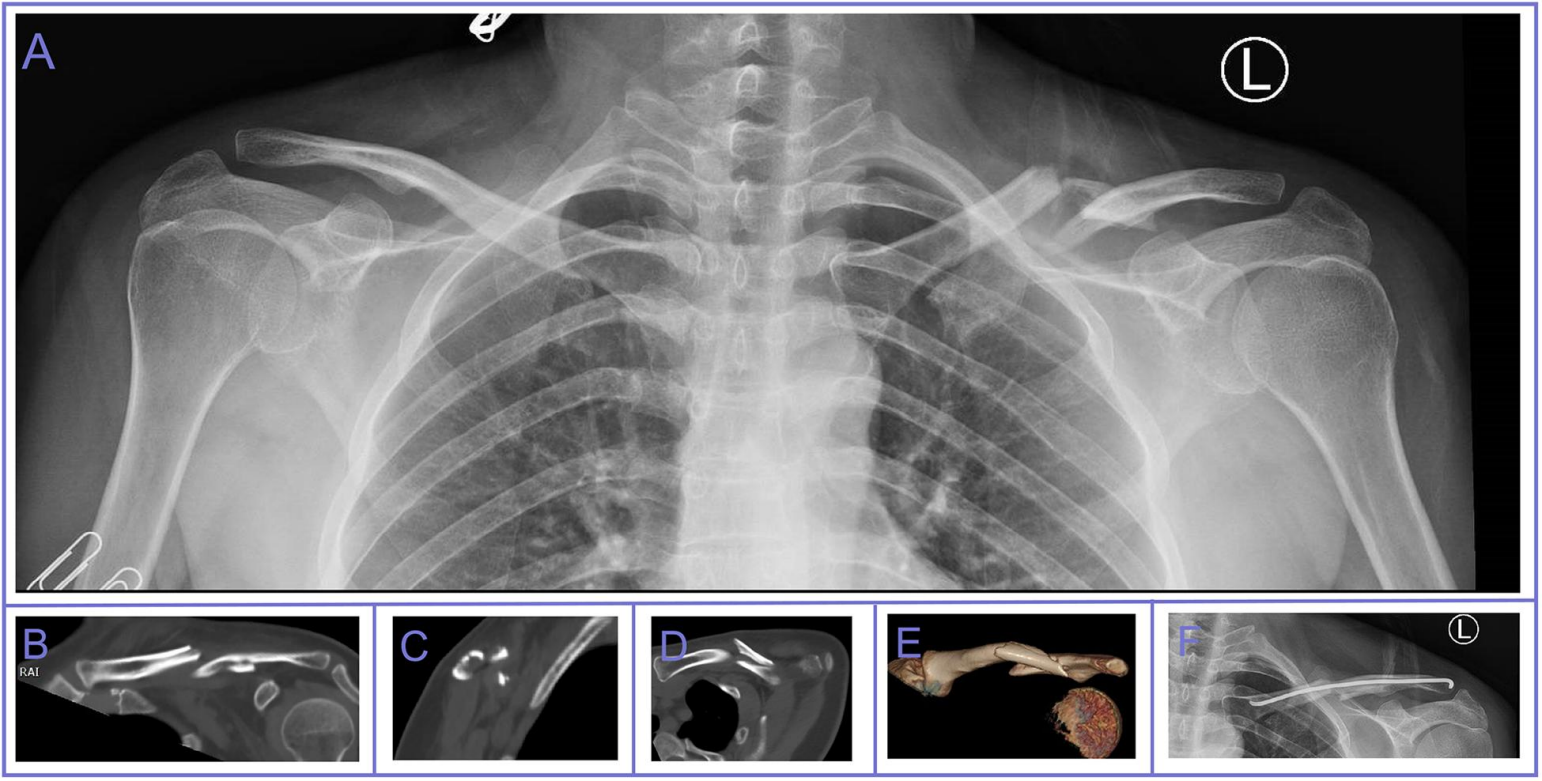
x-ray and CT images of the mid-clavicle fracture case. **(A)** Depicts the patient's initial post-injury anteroposterior radiograph, revealing a mid-clavicle fracture with significant displacement. **(B)–(E)** Present four different CT views, illustrating that the patient's clavicle fractures are more numerous and widely dispersed. **(F)** x-ray image on POD 1.

A 37-year-old woman presented to the emergency room on April 2, 2023, with complaints of left shoulder and chest pain. Five days prior, she had been involved in a collision with a tricycle while riding a motorized bicycle, resulting in mild dyspnea but no wheezing or other immediate discomfort. She did not seek medical attention immediately following the injury. Five days post-injury, she arrived at our emergency department, reporting persistent shoulder and chest pain. x-ray and Computed tomography revealed a completely displaced midshaft clavicle fracture and fractures of the left 2nd–5th and 7th ribs (Figure 1). She was subsequently admitted to the trauma department for surgical treatment of the displaced clavicle fracture, with conservative management for the rib fractures. Her medical history includes well-controlled diabetes mellitus managed with oral metformin and regular subcutaneous insulin injections. On physical examination, the patient's upper limb nerve function remained intact.

The patient underwent robot-assisted minimally invasive elastic intramedullary nail fixation for the left clavicle fracture (Figure 1F). The TiRobot assisted in accurately determining the posterolateral insertion point and direction of the distal clavicle, facilitating the placement of the elastic intramedullary nail (8). Closed reduction was performed, and the nail was manually advanced until it reached 1 cm medial from the articular surface of the sternoclavicular joint. Intraoperative fluoroscopy confirmed satisfactory alignment. The tail of the nail was bent and buried subcutaneously, and the incision was sutured. Following the operation, she was mobilized using an arm sling. From Postoperative Day 2, she was encouraged to engage in active exercise of the elbow and wrist joint on the same side, as well as passive exercise of the shoulder joint in a supine position. The arm sling was removed 2 weeks post-operatively then began rehabilitation under the supervision of a physiotherapist.

**Figure 2:**
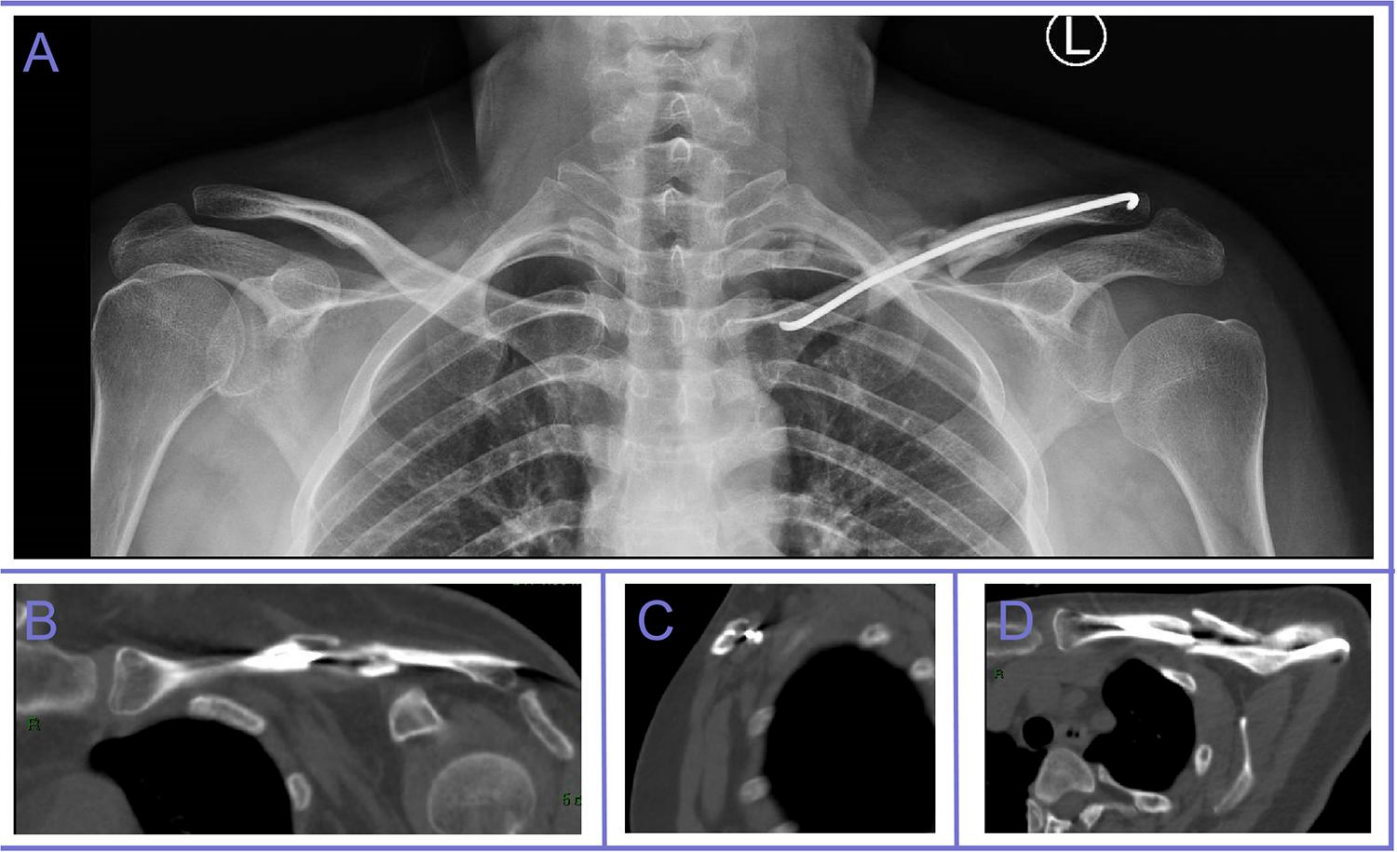
x-ray and CT imaging revealed significant bone callus formation at the fracture site.

On June 16, 2023, two months after surgery, the patient reported numbness in all fingers of the left hand, affecting both the palmar and dorsal sides, with a pronounced impact on the thumb. Additionally, the patient was unable to move the shoulder and elbow actively. During the physical examination, weakness in left shoulder abduction and elbow flexion was noted, with overall muscle strength in the left upper extremity reduced to grade 1 according to the Medical Research Council (MRC) scale. Additionally, decreased radial-palmar sensation suggested symptoms of median nerve injury. x-ray and CT imaging revealed significant callus formation at the fracture site (Figure 2). The electromyography (EMG) report of the supraspinatus, infraspinatus, biceps brachii, brachioradialis, and deltoid muscles shows a decreased number of motor unit action potentials during mild contraction, while during maximal contraction, the recruitment pattern is discrete in the supraspinatus, infraspinatus, and biceps brachii. Furthermore, the evoked potential latencies are prolonged in the supraspinatus, infraspinatus, deltoid, biceps brachii, and brachioradialis muscles. EMG analysis indicated severe damage to the C5 and C6 nerve roots, partially involving C7, C8, and T1. Doppler ultrasound identified thickening and reduced echogenicity in the upper trunk (0.53 cm), as well as the lateral and posterior cords of the left brachial plexus beneath the clavicle (up to 0.50 cm), suggesting structural changes in these areas. Based on the clinical, imaging, and electrophysiological findings, a diagnosis of left incomplete brachial plexus injury with root-level impairment was confirmed.

**Figure 3:**
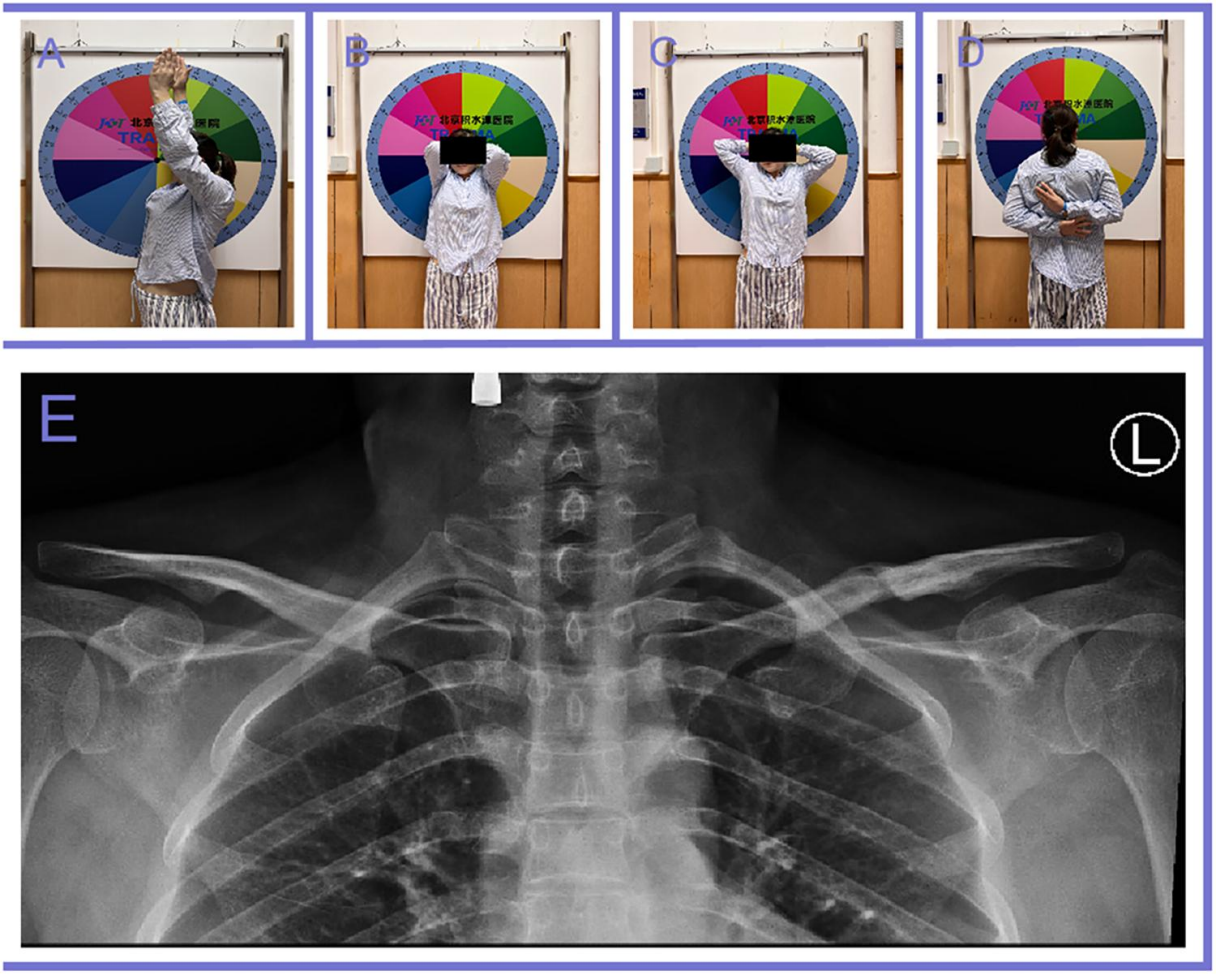
Good range of movement of the shoulders at the time of nail removal **(A)–(D)**. x-ray imaging after nail removal revealed notable bone remodeling with reduced callus formation at the fracture site compared to the findings observed at the onset of the brachial plexus injury **(E)**.

However, a brachial plexus injury specialist determined that there is no indication for immediate surgical exploration. Conservative treatment, including regular physiotherapist-supervised rehabilitation, was initiated. Over time, the patient experienced gradual improvement in muscle strength and resolution of numbness. 6 months after the operation, x-rays showed fracture healing. Eight months post-surgery, 6 months after the onset of brachial plexus injury, the patient requested the removal of the elastic intramedullary nail. At that time, a physical examination revealed improved shoulder abduction to 90°, with normal flexion and extension of the elbow, wrist, thumb, and other fingers (Figures 3A,B). Muscle strength in the left upper extremity had fully recovered to grade 5. x-ray imaging after nail removal revealed notable bone remodeling with reduced callus formation at the fracture site compared to the findings observed at the onset of the brachial plexus injury (Figure 3C). The patient reported an overall recovery of 95% compared to her condition at the time of the injury, reflecting a highly satisfactory outcome (Table 1).

**Table 1:** Timeline with relevant data from the episode of the patient.

Date	Timeline	Mainly results
2023-03-28	Traffic accident	Mild dyspnea
2023-04-02	Seek medical attention	Midshaft clavicle fracture and fractures of the left 2nd–5th and 7th ribs
2023-04-07	Surgery	Robot-assisted minimally invasive elastic intramedullary nail fixation
2023-04-27	First follow-up	The shoulder and elbow joints function well, and the sensation in the left upper limb is normal
2023-05-11	Second follow-up	The active movement of the shoulder and elbow joints is poor, while the passive movement is good. The sensation of the left upper limb is normal
2023-06-16	Third follow-up	Left incomplete brachial plexus injury with root-level impairment
2023-11-10	Fourth follow-up	The shoulder and elbow joints function well, and there is a slight loss of sensation in the left upper limb. x-rays showed fracture healing
2024-02-28	Fifth follow-up	The shoulder and elbow joints function well, and the sensation in the left upper limb is normal. The removal of the elastic intramedullary nail
2024-10-11	Telephone interview	Completely recovery

## Discussion and conclusions

Brachial plexus injury is a rare complication of clavicular fractures. In the acute setting, it is most commonly caused by direct compression from bone fragments. Lin et al. reported a case where brachial plexus compression occurred two weeks after the injury due to a vertically angulated intermediate fragment that narrowed the costoclavicular space; this was treated surgically (9). Similarly, Kim described a case of an acute displaced clavicle fracture with brachial plexus injury occurring three days post-injury, which was managed conservatively (3). Additionally, some authors have documented cases of brachial plexus injury immediately following intramedullary rod fixation or open reduction and internal fixation (ORIF) (4, 10). These cases did not identify definitive causes, but all were treated conservatively with good prognoses.

In delayed cases, brachial plexus injury manifesting weeks to months after the initial trauma is commonly caused by nonunion, malunited fragments, hypertrophic callus, or abnormal granulation tissue (11, 12). Saito et al. reported three cases where symptoms of brachial plexus palsy developed 27, 75, and 28 days after the initial injury due to abnormal granulation tissue formation (13). All three patients underwent surgical treatment (ORIF or clavicle resection) with favorable clinical outcomes. To the best of our knowledge, only one case of brachial plexus palsy has been reported to result from a nonunion clavicular fracture with extensive bridging callus formation following conservative treatment (6). In this instance, the patient underwent ORIF, which included extensive callus resection, local bone grafting, and brachial plexus debridement, leading to a good prognosis.

The patient in our case sustained a midshaft clavicle fracture and underwent robot-assisted minimally invasive elastic intramedullary nail fixation. Unlike ORIF, which involves exposing the fracture and may compromise the local blood supply—potentially leading to delayed bone union—intramedullary nail fixation helps preserve the local blood supply. In this case, the patient experienced numbness and weakness in the affected left upper extremity two months postoperatively. x-ray and CT imaging revealed significant callus formation at the fracture site, suggesting that the abnormal callus was the most likely cause of the brachial plexus injury. Previous studies have reported cases where extensive calluses and granulation tissues causing brachial plexus injuries were treated with ORIF and tissue removal (3, 6). However, based on clinical evaluation and other findings in our case, we concluded that the patient had an incomplete brachial plexus injury without the need for immediate surgical exploration. Conservative management was recommended, including rehabilitation exercises and acupuncture.

Over time, the patient experienced gradual improvement in muscle strength and resolution of numbness. 6 months after the onset of brachial plexus injury, a physical examination revealed improved shoulder abduction to 90°, with normal flexion and extension of the elbow, wrist, thumb, and other fingers. Muscle strength in the left upper extremity had fully recovered to grade 5. The patient reported an overall recovery of 95% compared to her condition at the time of the injury, reflecting a highly satisfactory outcome. To the best of our knowledge, this is the first reported case of brachial plexus injury following fixation with elastic intramedullary nailing. Our team has now performed a total of 208 cases of robot-assisted elastic intramedullary nailing of midshaft clavicle fractures. To date, only one case of postoperative brachial plexus injury symptoms has been observed. Additionally, it is worth noting that other complications have occasionally occurred, including 1 case of nonunion in a Type C fracture, 1 case of local infection, 6 cases of local skin irritation at the nail end, and 2 cases of skin breakdown.

Although brachial plexus injury following clavicle fractures is rare, it can be a serious complication with significant implications for recovery. The robot-assisted elastic intramedullary nailing technique for midshaft clavicle fractures offers a significant advantage by better preserving the local vascular network surrounding the fracture site (14). In contrast, conventional ORIF may compromise periosteal blood supply, thereby potentially delaying fracture healing. Nevertheless, according to our experience with over 200 cases, only one patient experienced a brachial plexus injury. Therefore, it would be inappropriate to infer that the robot-assisted approach increases the incidence of brachial plexus injury.

This case report describes a rare instance of delayed brachial plexus injury occurring two months after robot-assisted, minimally invasive elastic intramedullary nailing for a midshaft clavicle fracture. The injury was likely caused by the development of an abnormal hypertrophic callus, which exerted pressure on the brachial plexus. Despite the delayed onset of symptoms, conservative management involving rehabilitation and supportive care resulted in a gradual recovery, with the patient regaining full muscle strength and functional capacity. This case highlights the importance of vigilance for brachial plexus injury in patients with clavicle fractures and demonstrates the potential for successful outcomes with conservative treatment. Furthermore, it contributes to the limited body of literature regarding the rare occurrence of brachial plexus injury after elastic intramedullary nailing of clavicular fractures.

## Data Availability

The original contributions presented in the study are included in the article/Supplementary Material, further inquiries can be directed to the corresponding author.
